# The Nursing Supplement

**Published:** 1888-04-28

**Authors:** 


### The Nursing Mirror.

### En Passant.

THE PAY OF PRIVATE NURSES.—Every now and then the vexed question of the remuneration proper for nurses supplied to the public by institutions is raised. Most of these nurses earn from two to three guineas a-week, while the salary they receive seldom exceeds £30 a-year. The contention is that the institution levies too large a commission, and that though the sisterhood or hospital may be a worthy object of charity, still it is hard for the nurses to be forced to hand over to it a half of their incomes. It is certain that some of the private associations for supplying nurses have considerably benefited their founders, but from a purely business point of view this must be expected. The founder provided the capital and took all risks, and he expects a certain return. Then those who are connected with a sisterhood are supposed to devote their lives to charity and to be content with comparative poverty. Turning to the hospitals which now supply nurses for private cases, we find that their argument is that they have trained these women and actually paid them to learn, and that their surplus earnings for a few years after they are competent are only the due of the hospital. A probationer gets £10 or £12 during her first year for being of very little use, and her keep costs at least 10*s.* a week, not counting her share of rent, rates, taxes. wear and tear. When she joins the private staff she receives £30 a-year, her washing and travelling expenses, and her uniform. In the intervals between one case and another she is housed and fed ; and above all, her work is found for her, and she is saved worry and bother. Many a nurse who has started on her own acconnt has found that her larger earnings were more than balanced by her increased expenses ; and that while waiting for a fresh case she spent all received from her last patient. Institutions in connexion with the hospitals are all of recent date, and so far cannot have much more than covered the initial expenses which attend all new undertakings. Whether in the future they will see their way to having a staff of extra-mural nurses who will receive their own earnings and only pay a commission to the hospital is another thing. Such a scheme ought to be possible here, for it is common in many American towns. We gave an account of the directory of such an association in last week's issue.

WITH regard to the giving of presents by grateful patients, we think it is only right there should be a rule against it, as it does away with any doubt as to whether a nurse expects a gift or not. All such gifts might, however, be properly placed to the credit of those nurses who join the National Pension Fund, which would invest the money to the best advantage, and so greatly benefit the nurse. The sisterhoods generally demand that all substantial expressions of gratitude be handed over to their common fund ; the institutions place the money to the nurse's account on their pension list, and the hospitals sometimes permit the nurse to retain the gift, if the matron is satisfied it was unsought and the patient expresses a desire to present it to the nurse personally. Surely the trained nurse who wrote on this subject to the *St. James's Gazette* would not like the giving of presents to be the rule, except through the Pension Fund, for it is certainly derogatory to the dignity of her profession. The earnings of women are disgracefully small, whatever work they take up, but this is because they seldom have any business training, and cannot usually manage their own affairs. It is hard for nurses to be so poorly paid, but their remuneration is increasing yearly, and presently when they better understand provident and business habits, they will be able to pocket a fairer share of their earnings. Nurses, more than other people, need to keep free from personal worries, that they may minister with absolute unselfishness to the sufferers under their charge ; and if they are constantly thinking where they will get another case, and writing letters and answering applications, they will find the strain too great. Better a small salary and peace of mind, than larger earnings and worry therewith.

THE LANCET.—When you find your point untenable, it is rather a good plan to suddenly take your stand on an absolutely opposite point. Thus, the *Lancet* having discovered one week that the Pension Fund charged higher rates than any great insurance companies, and having had this point disproved, wisely changed its ground, and last week argued how derogatory it was for nurses to avail themselves of a charitable undertaking like the Fund, which gave them more than they could buy with their money elsewhere. How the Pension Fund is going to swindle nurses out of their earnings, and bestow degrading charity on them at the same time, we do not quite understand.

EXAMINATIONS.—The time for the annual examinations of probationers in the large training schools is drawing near. Many a nurse's heart fails her as she thinks of the dreaded ordeal, for if she fails to pass and secure her certificate, there will be small hope of obtaining a lucrative post in the future. We hope to publish at intervals specimens of examination questions given to probationers, that those who desire it may prepare themselves by trying to answer them, and so become familiar with the style of test likely to be demanded by the examiners.

NURSING IN CANADA.—A nurse writes to us to say that the Secretary of the Women's Protection Emigration Society assures her that there is every prospect of success in Canada for sober, efficient nurses. The Secretary meets emigrants on their arrival at Montreal, and the Society does all in its power for them. The pay for nurses is one dollar a day for non-infectious cases. Our correspondent would be glad to hear of other nurses wishing to go to Canada in May or June. The following are the exact words of the communication we received from the Canadian Government on the subject : "In reply to your letter of the 31st ult., I beg to say that I am not aware of any nursing institution in Canada such as you mention. No doubt a few well-trained nurses would always be able to get employment, but we do not encourage persons of that class to go out unless they have friends in Canada, or have by previous communication become assured of the probability of securing an engagement on their arrival."

TWENTY PER CENT.—We have had forwarded to us the rules of a certain Nursing Staff, which proclaims its hope of procuring agents in every town. We fancy this hope is too sanguine, for surely many nurses will not join an association of which the following is a rule : "Non-resident nurses on this staff are charged an inclusive commission for introducing the appointment, &c., at the rate of 20 per cent., or 4*s.* in the pound per week, upon the gross amount of remuneration obtained. If a nurse obtains an engagement for three weeks at £1 per week, the commission charged would be 12*s.*, but never less than 5*s.* is accepted as commission in any single engagement."

HOSPITAL NURSES IN INDIA.—Miss Oxley, lady superintendent, and the Misses White, Beresford, and Muller, lady nurses, have arrived from England for duty in the military hospitals in the Bangalore station. Dr. Benson, Durbar Physician, has succeeded in getting six girls from the Wesleyan Mission Orphanage to be trained as nurses in connexion with the Lady Dufferin Fund. This is a good beginning, and we wish the experiment a triumphant success.

### The National Pension Fund for Nurses.

### SPECIAL NOTICE TO NURSES.

It does not appear to be generally understood that all nurses under fifty years of age, who wish to participate in the benefits of the Pension Fund, are invited to join the Fund by paying into it one-eighth of their present earnings per annum. All nurses who really desire to be helped to the utmost to help themselves, and whose circumstances are such as to prevent them from putting by a larger sum than one-eighth of their income, are invited to apply to the Secretary, 38, Old Jewry, E.C., for a special form of application, which should be filled up and returned with as little delay as possible. Those nurses who have the foresight to adopt this course, may rest assured that their applications will be most carefully considered, with the view of securing them an adequate provision when overtaken by permanent illness or old age.

### HONEST CRITICISM OR VINDICTIVENESS?

There has been a practical unanimity among leading journals of the first-class as to the great value of the National Pension Fund for Nurses. Among those which have—some of them several times—expressed approval may be mentioned: *The Times*, *The Standard*, *The Daily News*, *The Daily Telegraph*, *The Daily Chronicle*, *The Morning Post*, *The St. James's Gazette*, *The Pall Mall Gazette*, *The British Medical Journal*, *The Medical Press and Circular*, *The Illustrated London News*, *The Echo*, *The Financial News*, *The Dublin Express*, *The Birmingham Daily Post*, and others.

Against this consensus of approval there is to be set one weekly medical newspaper. This print is to be challenged on its facts. It avows that there are old-established insurance offices which will provide pensions for nurses at cheaper rates than the National Pension Fund provides them. The obvious answer is—Name the insurance offices. If the offices are not named after this challenge, it will be fair to infer that it is because there are no such offices.

A smaller paper, recently issued, first declares that the benefits of the Fund are of no value to nurses because they are too costly; and then, with truly feminine inconsistency and inconsequence, scolds the promoters for offering a share of the benefits to "those most charming officials the secretaries," as the writer with gentle suavity, describes them. If the benefits are really injurious to nurses, surely the people who love secretaries so well as to dub them in a public print "those charming officials," need not grudge them an *injurious benefit*. Do these papers venture to assert that the yearly interest on £26,000 will be an injury to nurses?

For my part, I should at least respect the candour of both these journals if they would honestly say they hate the founder of the Fund so cordially that they will not allow any work he does to prosper if they can help it. Their present behaviour calls to mind the conduct of fierce and ignorant Irish peasants who, because they hate their landlord, burn down his hay-ricks, hamstring his horses, and mutilate his cattle. Such conduct has earned at least one man's contempt.

Diogenes.

### WHAT THE NURSES THINK OF THE FUND.

H. N., writing from the Hertford British Hospital, Rue de Villiers, Levallois-Perret, Seine, says: "I do not think you will have to wait long for the thousand nurses to join your Pension Fund. I believe they will be only too glad to accept such liberal offers. I can answer for myself, for I hope to join it before long."

[We should be glad to hear the views of other nurses.—Ed. *T. H.*]

The Case of the Older Nurses.—Another correspondent points out that the premiums, as set forth in the tables printed in the prospectus, though very favourable to the younger nurses, are found almost prohibitory to those who are forty years of age or upwards. This is no doubt true if they have put nothing by already, and yet desire to secure a minimum of 10*s*. per week pension. What we advise such nurses to do is to join the Fund for a smaller pension, and to write to the Secretary a full statement of their difficulty and case. If they take this step they may rely upon the Council to secure them the utmost allowance possible, and to take care of them in the day of their necessity. It should be clearly understood that those nurses who join the Fund during the first year will have the first and strongest claim for consideration when the Bonus Fund is distributed.—Ed. *T. H.*

Mrs. Bedingfeld's letter is unavoidably held over until next week.

### APPLICANTS' DIFFICULTIES.

[All questions should be sent to the Secretary, the National Pension Fund, 38, Old Jewry, E.C.]

Questions asked by Applicants and Answers Sent.

*(Continued from p. vi.)*

11. Can a nurse recover the money she has paid into the Fund any time before the pension is entered upon?

Yes, if she joins under the returnable table.

12. What premiums would a nurse of 30 have to pay to receive £50 a year at the age of 50 and 20*s*. sickness allowance?

Taking age at 31 next birthday, the following are the quarterly contributions, to cease at 50, when the benefits will commence: Annuity (£50 per annum), £8 13*s*. 4*d*.; sick pay (20*s*. per week), 6*s*. 9*d*.; total. ££9 0*s*. 1*d*.

13. Can a nurse receive her pension if resident abroad?

The sick pay will cease on her permanently leaving the United Kingdom, and the contribution for it will cease also. She may continue her contribution for the annuity, and draw the annuity abroad when it becomes due.

14. On what terms could a nurse 42 next birthday purchase an annuity of £1 per week for life from age 50 (returnable)?

This nurse would only pay premiums for eight years, whilst she may reasonably hope to have a pension of £50 per annum for at least 21 years, yet her quarterly contributions would only be £12 14*s*. 8*d*.; single payment, £357 9*s*. 6*d*.

15. What immediate annuity would be received by a nurse 48 next birthday for a single payment of £300?

£17 6*s*. 8*d*. per annum for life.

16. What sum should be paid by a nurse aged 50 to secure a pension of 10*s*. a week at 60?

The single payment is £259 4*s*. 10*d*., returnable; £220 17*s*. 10*d*., not returnable.

17. What pension could a nurse, 46 next birthday, obtain for a payment of £300 now, and £100 two years hence?

A payment down of £300 would secure a pension of £19 11*s*. 6*d*., to commence at 50; returnable in case of previous death or withdrawal. Two years hence another payment of £100 will secure an additional pension of £6 5*s*. 4*d*. on like conditions.

18. What pension would the payment of a single sum of £300 secure at the age of 60 for a nurse who is 53 years old?

A pension of £27 11*s*. The £300 would be returned if applied for before the age of 60.

19. What will be paid at once by a nurse, 41 next birthday, who desires to enter as of 40 years of age?

Under Table IV., the sum to be paid at once is £17 3*s*. 4*d*., if rate for the age of 40 is to be paid.

20. Will yearly contributions, paid in advance, be accepted at reduced rates?

No.

21. What extra sum must a nurse (29), who has taken out an annuity of £22 10*s*. at 50, pay to receive a sickness allowance of 10*s*. a week?

Assuming age 30 next birthday, the quarterly payment for a sick allowance is as follows: To cease at 50, 3*s*. 4*d*.; to cease at 55, 3*s*. 9*d*. These are, of course, not returnable.

### A District Matron's Diary.

[Contributions for this column are cordially invited.]

We are enabled, by the courtesy of the writer, to lay before our readers a few pages from the diary of the matron of the East London Nursing Society. The matron has several parishes under her charge, but tries to do the round of each parish every week, or oftener. The following notes all refer to the same parish, and they will give a fair idea of the character of the work done and of its immense value to the poor:—

*February 15th.*—Four new cases. Mrs. W., cancer; at present nurse does little for her, but Dr. C—— begs her to go, as he intends ordering syringing soon. Mrs. A—— burst a blood-vessel on the brain. The poor woman is very ill indeed, and needs careful watching. Mrs. L——, severely ulcerated legs. Nurse found her extremely dirty, the rags positively growing into the wounds, so long had they been neglected. Her room, bed, and person were in the same state. Bed-clothes and body-linen have been sent, also a doctor, and a woman to clean her room, prepare her food, &c. The fourth is a case of phthisis, which we hope to remove to the hospital to-morrow. The man H. is still weak and in bed; I fear the mischief is in the head more than the body. Mrs. J. has recovered. Mrs. W. and others much as usual.

*February 23rd.*—I have had a long round to-day. Mrs. W. is in bed and feeling very weak and often in great pain; she needs a mackintosh, which nurse will take to-morrow. Mrs. A. looks extremely ill, and must be kept quite quiet for a time. I am glad to say there has been no return of the dreadful retching which caused the blood-vessel to burst. Nurse is very attentive to her. Mrs. L., whose legs are badly ulcerated, I find much improved in every way; her room clean, and her legs carefully dressed with zinc ointment. H. is really getting better, I hope; so much has been done for him both in the way of nursing and of nourishment that we eagerly look for a good result. B. and P. are much improved, and can come off the register. The child C. has lost all bronchitis; her disease now looks like phthisis, she is so helpless and has no appetite.

*March 2nd.*—Mrs. W., suffering from paralysis; her case has been a very severe one, but she has a little feeling coming into the left hand, and I think she will recover this stroke. W., a lad suffering from acute pleurisy, poultices are applied. A very old woman is dying of decay; she is as helpless as a baby, and nurse keeps her most beautifully clean. H. is not so well; very restless all night. The child C. looked brighter and better. Mrs. A. had one of her bad days. The rest much improved.

*March 6th.*—One new case—Baby C., whooping-cough and bronchitis; poultices are applied and medicine given. The poor old woman S. is still alive, but quite unconscious. Mrs. W., a new case, suffering from pleurisy; poultices are applied. The boy W. is rather better to-day; I am sending flannel shirts. The child C. looks better, the cotton wool still on chest and back; I am sending flannel chemises, that it may be left off. Mrs. A. is allowed to sit up a little each day, and is now out of danger. Mrs. M. looked very weary when I went in to see her, but she brightened wonderfully; she is always so grateful for a visit. Mrs. L.'s ulcerated legs much better. Mrs. W., case of paralysis, still improves. The boy W very ill again; another abscess forming. H. is better to-day than I have seen him, owing to a good night. I had a note from Dr. C. yesterday begging me to send help at night; he will be pleased at the improvement. Nurse is not very well.

*March 14th.*—Two new serious cases: Mrs. C., abscess of breasts; poultices and hot fomentations. Her child is much better; she was very anxious to show me she had on my flannels, and that she had not destroyed the picture-book I gave her. Mrs. A—— has recovered, and is going to a convalescent home. The poor old woman S. has gone to her rest. The boy W. goes to a convalescent home next week. The boy W. is again out of pain, and as well as usual; nurse will be required if another abscess forms. Baby C. is still very ill. Mrs. H. is the other new case; she has dropsy and other internal troubles. I saw this case with Dr. C——, who is trying medicine before he decides to tap her. I found poor H. in great pain; I called to see nurse, and asked her to give a simple enema; as I met Dr. C—— after, I confessed my interference; he said I was right, which was a relief. Nurse is still used up; I must get help for her.

*March 21st.*—Two new serious cases. H. W., lacerated leg, a kick from a horse while trying to help it to rise after a fall; he is a very nice boy, and anxious to get to work again to help his mother, who is a widow. Poultices were first applied, and now lotion; the wound looks healthy. His mother was washing in the most contented way, not in the least knowing where coals would come from to keep up her copper fire. I ordered a little coal, which surprised and pleased her very much. The next is a case sent me by Dr. C.—Mrs. W.; she is suffering from hæmorrhage; she also is a widow, and works very hard to keep her family. I sent for beef-tea and milk, which were ordered by the doctor. The poor woman looked very weak. Poor H. has had a relapse, and I am very fearful, after all our care, that he will not pull through. The child C. is well.

*March 27th.*—A letter from Dr. C. asking me to see the case H., to give him my advice about moving him to the hospital as a last chance. He fears cancer of the bowel. H. seemed better to-day, and after a little talk with him and his wife, he consented to go, saying he felt equal to being moved. I then went to nurse's lodging to tell her to make arrangements about an ambulance, and from there on to Dr. C., to tell him what I had done. I am very disappointed about this case, so much has been done, and it is very sad for the family. Mrs. W. is still very weak, though the hæmorrhage has stopped. Mrs. C. has recovered from abscess of breasts. The boy W.'s leg is much better.

*April 2nd.*—The man H. has been safely removed to the hospital. This is a great relief, as the case was a most anxious one, and needed constant attention. The woman L., whom nurse found so dirty and neglected, and did so much for, as soon as she could get out got drunk and was much worse than before. She was removed to the infirmary, and £200 were found in the dirty rags in odd corners of her room. I hear she has just died at the infirmary.

### Anecdotes of Personal Adventure.

I was sent to a case in co. Kerry, and found my patient hopelessly ill. The family were very noisy in their grief and very inconsiderate to me, but since I knew the case must be a short one, I consented to being on duty night and day, and only snatching an hour's sleep now and then. The food was very bad, and there was no bath in the house; still, I bore everything uncomplainingly for five days, when my patient died. I performed all the last offices, and thoroughly wearied out, was about to retire to my room, when I was met by the mother of the deceased, who insisted that it was my duty to sit up all night in the death chamber. I argued with her in vain on the uselessness of this form; she would not be content till she had supplied me with a large stock of wax candles and shut me in with the dead. Another time, nursing in a titled family in the north of England, I was eating my dinner in the housekeeper's room, when the butler entered, and asked if I would like a glass of champagne. I replied that I was a teetotaller; but, with a wink he produced a bottle from behind his back and declared that if I would but kiss him I should have as much as I liked, and he would never tell a soul.—*Nurse Gertrude.*

### Appointments.

Miss Augusta Forbes has been selected out of upwards of seventy applicants for the post of matron to the Hospital for Sick Children, Paddington Green. Miss Forbes is 32 years of age, and has had six years' experience in nursing children. She was formerly a nurse and afterwards matron of the Aberdeen Children's Hospital, in which Dr. Sarden takes so deep an interest; and subsequently she had charge of two wards as sister, serving afterwards for one year as out-patient sister at Great Ormond Street. Miss Forbes' testimonials are of a very high order, and whilst we congratulate the committee on their appointment, we hope that the new matron will find the Paddington Green Hospital not only a comfortable home but a most profitable and pleasant field of labour.

### Lectures.

April 27th, at 3.30, at 1, Carlton Gardens, Mrs. Ayrton on "Women of Science." Admission, 7*s.* 6*d.*

May 1st, at 3 p.m., at the Royal Institution, Walter Gardiner, on "The Plant in the War of Nature." Three lectures, 10*s.* 6*d.*

### Everybody's Opinion.

### A CASE FOR THE PENSION FUND.

L. W. writes: You may be surprised to hear from me again, but I read The Hospital, and hear a great deal about the Pension Fund. I do so wish it had been formed years ago, before my health broke down. For three years I have been paralysed, but am better now, and hope to soon get to work again. If I could only get a few weeks by the sea I am sure I should recover my strength. As soon as ever I can commence nursing once more I shall join the Fund if they will have me.

### THE BRITISH NURSES ASSOCIATION.

In your last issue "A Reader," in reply to a letter of mine, accuses me of trying to prejudice nurses from joining the above Association. I may have formed a wrong judgment of the Association, but certainly had no preconceived opinion about it before reading the rules, nor did I wish to influence others, but rather to procure the enlightenment of my own ignorance. I was not able to be present at the meeting mentioned by "A Reader," but from perusal of printed bye-law No. 4 of the Association, I certainly understood that till January 1st, 1889, any nurse who had worked three years, whether in a hospital or not, was eligible to become a member. If such is not the meaning of the bye-law it ought to be rewritten, for the present wording is distinctly to that effect.—F. H. M.

### Notes and Queries.

To Correspondents.—1. Questions may be written on post-cards. 2. Advertisements in disguise are inadmissible. 3. In answering a query please quote the number. 4. If a private answer is desired, a stamped addressed envelope must be forwarded.

(6) *A. M.* would like to know if there are any institutions for invalids that would give a trained nurse cases in London, so that when not engaged she could reside at home till required.

(7) *Tabitha* wants to know if a lady who has only trained six months in a hospital, would be considered competent to take holiday work in a cottage hospital.

(8) Where can I procure air-balloons in the City? I have a children's ward where this cheap toy is much appreciated, but can seldom procure it. —*Sister Child.*

(9) One of the advertisers in this journal has sent us the following letter, just received, wishing to know what it means?

The British Gynæcological Society, April 1888.

Sir,—The journal of the *British Gynæcological Society* is now published by Messrs. John Bale, 87, Great Titchfield Street, W. In future, advertisements should be sent to Dr. Bedford Fenwick, 20, Upper Wimpole Street, W. I am pleased to be able to inform you that the circulation of the journal has increased by 300 copies over that of last year.—Yours faithfully,

The Editor.

(10) *E.* would be glad to know whether it is the custom in cottage hospitals where there is a matron and one servant for the women patients constantly to frequent the kitchen?

**Answers.**

(1) *L. W.* (Leicester).—You can join the Pension Fund at any time.

(2) *M. H. L.*—If your absence is only temporary you will be eligible to join the Fund.

*Miss B.*, West Brighton, writes: Will you kindly send address of the Nursing Institution in Philadelphia, mentioned in the The Hospital this week?—The Directory of Nurses, Philadelphia, U.S.A.

### Vacancies.

The following vacancies are announced:—

**Matrons.**

Blackburn Infirmary; £80.

**Nurses.**

Royal Hospital, Portsmouth; £25.

Stroud Hospital; £23.

Rochdale Infirmary; £27.

Hemel Hempstead Infirmary; £22.

Nottingham Nursing Institution.

Victoria Home, Bournemouth.

Convalescent Institution, Woolton; £20.

Miss Boor's Home, Nottingham; £25.

Worthing Infirmary; £20.

Jersey Institution; £25.

Children's Home, Sheffield; Night.

Victoria Home, Bournemouth; Several.

Birkdale Home, Southport; Several.

Hospital Edinburgh Poorhouse, Thoroughly trained; £30 to £35. Ordinary trained; £25.

**Probationers.**

Hospital for Women, Liverpool.
Blackburn Infirmary.

### MR. BURDETT'S BOOKS ON HOSPITALS.

"Mr. Henry C. Burdett, whose interest in Hospitals is well known, is widely recognised as the chief authority on the subject."—*The Lancet*, July 25th, 1885.

### HOSPITALS AND THE STATE: HOSPITAL MANAGEMENT, AND HOSPITAL NURSING.

With Statistics of the Chief Medical Institutions in Great Britain and Ireland. [Cloth, 2s. 6d.

*Boston Medical and Surgical Journal.*—"Mr. Burdett is one of the highest authorities of Great Britain on the subject of Hospital Administration."

*British Medical Journal.*—"The only accurate statistics of hospital income and expenditure, prepared upon an identical basis, which have ever been published."

### COTTAGE HOSPITALS: GENERAL, FEVER, AND CONVALESCENT.

Their Progress, Management, and Work. With an Alphabetical List of every Cottage Hospital at present opened, and Chapters on Mortuaries, Convalescent Cottages, and the Relative Mortality in Large and Small Hospitals. The book contains the ground-plan and elevation of the best constructed British Hospitals and Medical Institutions having fifty beds and under, and also a Portrait of Albert Napper, Esq., the Founder of Cottage Hospitals. Second Edition. 600 pp. [Cloth, 14s

**SELECTED EXTRACTS FROM REVIEWS.**

*Birmingham Medical Review.*—"An excellent book, full of practical information."

*British Medical Journal.*—"A sensible, handy, and complete work. . . . Contains everything which could be required by anyone who undertakes to found or to manage a cottage hospital."

*Medical Times and Gazette*—"A good work, and cannot fail to be of value."

*Sanitary Record.*—"We do not hesitate to say that, as a text-book of practical reference, it ought to be in the hands of everyone interested in hospital management, and it is gratifying to know that this opinion is shared by a very large section of the medical profession."

### PAY HOSPITALS AND PAYING WARDS THROUGHOUT THE WORLD:

Facts in support of a Re-arrangement of the English System of Medical Relief. 200 pp. [Demy 8vo., 6s.

**SELECTED EXTRACTS FROM REVIEWS.**

*British Medical Journal.*—"Mr. Burdett is well known to the medical profession as an advocate of provident dispensaries and hospitals, and as the originator of the Home Hospitals Association. All who are interested in these subjects will be gratified by this volume. In it the author deals with the whole question of pay hospitals and dispensaries in a large and comprehensive spirit. We commend the book to all who are interested in the improvement of medical relief—and which of us is not? It is clearly and pleasantly written, it is full of facts, and it contains many valuable suggestions. This work cannot fail to stimulate the reforming movements which have been gathering strength in this country during the last few years."

*New York Medical Journal.*—"Mr. Burdett has devoted many years to the study of hospital administration and management, and his book and plans are well worth perusal."

*The American Practitioner.*—"Mr. Burdett displays and discusses the whole scheme of hospital accommodation with a comprehensive understanding of its nature and extent, and he does it in fulness without prolixity, and in a clear catholic spirit with perspicacity. The book is a stepping-stone, a valuable contribution in the way of introduction to a review and candid reconsideration of the whole subject of legal and organised charity—a theme which much demands consideration and re-adjustment in the whole civilised world. A good and timely book, and suggestive."

London: J. and A. CHURCHILL, 11, New Burlington Street, W.

